# In-line Raman imaging of mixing by herringbone grooves in microfluidic channels†

**DOI:** 10.1039/d4lc00115j

**Published:** 2024-06-17

**Authors:** W. J. Niels Klement, Elia Savino, Wesley R. Browne, Elisabeth Verpoorte

**Affiliations:** a Molecular Inorganic Chemistry, Stratingh Institute for Chemistry, Faculty of Science and Engineering, University of Groningen Nijenborgh 4 9747 AG Groningen The Netherlands w.r.browne@rug.nl; b Pharmaceutical Analysis, Groningen Research Institute of Pharmacy, University of Groningen Antonius Deusinglaan 1 9700 AD Groningen The Netherlands

## Abstract

The control over fluid flow achievable in microfluidic devices creates opportunities for applications in many fields. In simple microchannels, flow is purely laminar when one solvent is used, and hence, achieving reliable mixing is an important design consideration. Integration of structures, such as grooves, into the channels to act as static mixers is a commonly used approach. The mixing induced by these structures can be validated by determining concentration profiles in microfluidic channels following convergence of solvent streams from separate inlets. Spatially resolved characterisation is therefore necessary and requires in-line analysis methods. Here we report a line-focused illumination approach to provide *operando*, spatially resolved Raman spectra across the width of channels in the analysis of single- and multi-phase liquid systems and chemical reactions. A scientific complementary metal oxide semiconductor (sCMOS) sensor is used to overcome smearing encountered during spectral readout of images with CCD sensors. Isotopically labelled probes, in otherwise identical flow streams, show that *z*-confocality limits the spatial resolution and certainty as to the extent of mixing that can be achieved. These limitations are overcome using fast chemical reactions between reagents entering a microchannel in separate solvent streams. We show here that the progression of a chemical reaction, for which only the product is observable, is a powerful approach to determine the extent of mixing in a microchannel. Specifically resonance enhancement of Raman scattering from a product formed allows for determination of the true efficiency of mixing over the length and width of microchannels. Raman spectral images obtained by line-focused illumination show onset of mixing by observing the product of reagents entering from the separate inlets. Mixing is initially off-centre and immediately before the apex of the first groove of the static mixer, and then evolves along the entire width of the channel after a full cycle of grooves.

## Abstract 2

In-line characterization of mixing in microchannels with high spatiotemporal resolution is challenging. Here we describe a Raman imaging approach to ascertain extent of mixing in across channels in 3D for both same- and immiscible-solvent systems.

## Graphical abstract

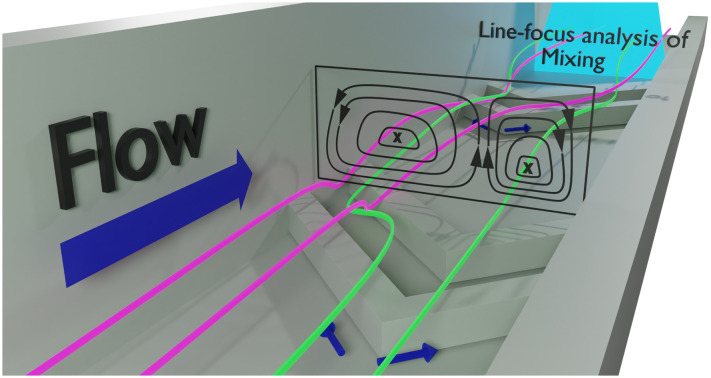

## 1 Introduction

Microfluidic devices are employed widely in chemistry and biology with applications as diverse as photochemical flow reactors,^1^ organ-on-a-chip systems,^2^ (bio-)analytical chemistry,^3–6^ cell sorting^7^ and Covid19 detection.^8^ In many systems, the efficient mixing of fluid streams in microchannels is essential. This is especially the case when chemical reactions are desired, as molecular reagents need to collide to react with each other. In a simple microchannel with smooth walls, fluid flow is purely laminar at the flow rates most often used in microfluidics. Mixing of components between streams flowing side-by-side in the laminar regime is dependent on diffusion. Where mixing is diffusion-controlled, the overall time for a reaction to reach completion (*i.e.* the residence time needed in the channel) depends on channel dimensions, as they dictate diffusion distances. This limitation can demand channels that are unacceptably long, or flow rates that are too low for desirable throughput.^9^ Furthermore, for reactions with high reaction rate constants, the reliance on diffusion for mass transport can lead to a large variation in observed reaction rates across the channel width.

**Fig. 1:**
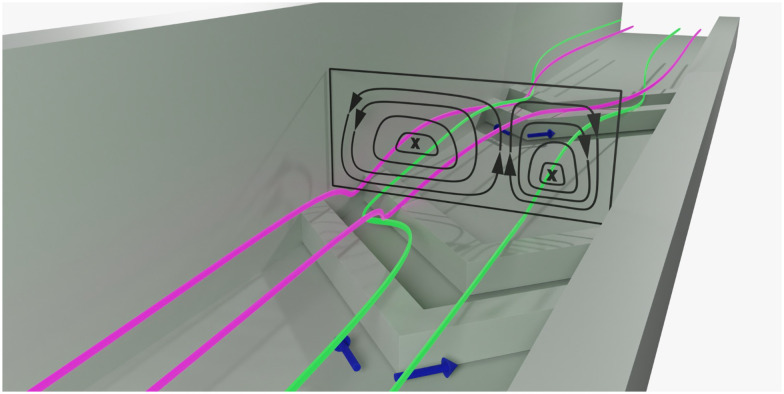
Microchannel with slanted herringbone grooves. Liquid streams flowing side by side tumble over the grooves. The slanted geometry of the grooves result in vortices which disturb the laminar flow.^10^ The flow profile inside microchannel is shown as a cross-section. Mixing in this flow pattern still relies on diffusion. However, vortex formation ensures displacement of the each solvent stream across the channel cross section, increasing contact between them decreasing the distance over which diffusion needs to take place with respect to unperturbed laminar flows, where species must diffuse across a single contact plane. Hence the mixing time is decreased overall.

Mixing on a molecular level is always diffusion-based and thus a passive process. Convection increases observed mixing rates by substantially increasing the contact area between the solution streams containing the reagents. This shortens the pathlengths that reactive species have to traverse by diffusion in order to react with each other.^10,11^ Introducing structures (static mixing elements), such as groove arrays, to perturb laminar flow and create chaotic flow profiles within microchannels is a well-established approach to minimize gradients in reaction rate and mixing times.^9,10^ Chaotic micromixer designs of many shapes and sizes have been described, each with their own particular characteristics.^9^ One of these designs, the slanted herringbone mixer (SHM, Fig. 1), has found many applications due to the high mixing rates achievable over an enormously large range of flow rates (5–1000 μL min^−1^ for a 300 μm-wide channel).^9,10^

Computational fluid dynamics (CFD) is used widely to model flow in microchannels containing various designs of grooves for mixing, and allow optimization of structure geometries and dimensions to aid experimental implementation.^12–14^ Additionally, CFD can help visualise the expected flow profiles inside channels.

Several experimental approaches have been developed to characterize mixing in channels.^4,12,15–17^ In-line measurements, performed in the channel during operation, analyze changes in real-time. Off-line analysis investigates products that exit the channel. Both approaches have their merits; however, off-line analysis inherently loses resolution in time and distance, which may yield an imprecise view of mixing characteristics. In-line analysis is better suited for measurement of mixing characteristics, as mixing patterns evolve over distance in the channel. The in-line methods available are most commonly based on fluorescence imaging and single point scanning, and both have been applied to characterise the flow profile induced by SHMs, Fig. 1.^9,10^

Spectroscopic imaging using Raman scattering has received increased attention recently as it is a so-called label-free technique; Raman scatter originates from solvents and analytes directly, omitting the need for additional reporting components (*e.g.*, fluorophores).^18–20^ As with fluorescence spectroscopy, a Raman spectrum can be obtained non-invasively, which makes (imaging) Raman spectroscopy well-suited for in-line measurements. A further advantage of Raman spectroscopy is that the spectrum obtained generally yields multiple sharp bands, often referred to as a molecular fingerprint, enabling precise identification and quantification of each component in the solvent stream. Examples of applications of in-line Raman spectroscopy include in-line reaction monitoring, and identification and sorting of cells.^19,21–24^

Raman spectroscopy is limited to some extent by the inherent weakness of Raman scattering.^20^ Techniques based on ultrafast laser excitation, such as coherent anti-Stokes Raman spectroscopy (CARS)^25,26^ and stimulated Raman spectroscopy (SRS)^27^ are able to overcome this limitation to a considerable extent. Moreover, the availability of low noise (electron-multiplied) charge-coupled device ((EM)CCD) detectors has allowed for low-power, continuous-wave, laser-based Raman spectroscopic imaging with high signal-to-noise ratios.^28–30^

We use here resonance enhancement to increase the sensitivity of Raman spectroscopy to solutes present at low concentration. It requires only that the wavelength of the laser used coincides (is resonant) with an absorption band of the dissolved component of interest.^31^ Solutions containing compounds with large conjugated π-systems or transition metal complexes often satisfy this criterion. Resonance enhancement can increase limits of detection by two to six orders of magnitude.^31–33^

Conventional confocal fluorescence microscopy builds up images by rastering repeatedly over a region of interest with a single-point focused laser to acquire spectra sequentially from each point.^10,34^ In state-of-the-art scanning systems, illumination for as little as 0.25 s at each point (pixel) is sufficient to obtain an image.^30^ However, even for a few tens of points, the acquisition times become quite long (seconds to minutes) with this approach. Besides rastering the laser over a sample, imaging can also be achieved by moving the sample with respect to the laser spot. A drawback in both cases, though, is the need for movement.

An alternative approach to achieving spectral imaging in one dimension is to use a laser with a highly elliptical beam profile, which approximates a collimated rectangular cross-section, such that the illuminated object in the optical system is a line rather than a point. The Raman scattering collected in the confocal (cylinder) volume is then imaged onto the detector as a line rather than a spot also, with each row of the detector corresponding to a different height in the object plane (*e.g.*, a microchannel cross-section). Therefore, neither the sample nor the laser needs to be moved. An additional advantage is the reduction in power density, which is especially useful in high-power applications such as nonlinear imaging systems.^35^

**Fig. 2:**
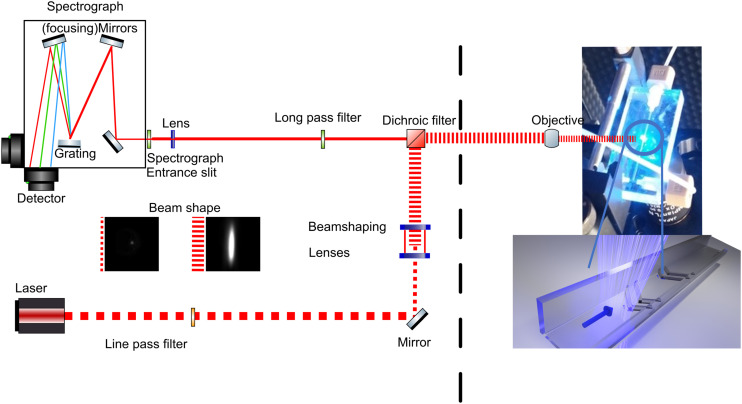
Line-shape illumination on a microchannel, spectrometer layout, and optical arrangement used in the present study. The beam is expanded into an elliptical shape after the beam shaping lenses. The beam is then directed onto a microchannel (photograph), which results in a sheet of light over the width of the channel. The collection of Raman scattering is shown as a solid line. Images of the beam profile with and without beamshaping are shown as an inset.

**Scheme 1:**
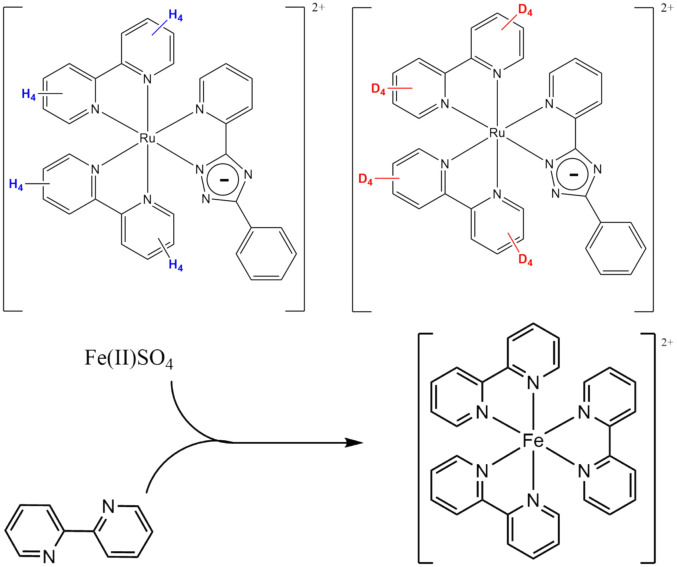
(Top) Isotopically labelled ruthenium(ii) complexes used for Raman imaging of mixing of solvent streams, and (bottom) reaction between the colourless 2,2′-bipyridine and iron(ii) sulphate, to produce the red-coloured [Fe(bpy)_3_]^2+^. See Fig. S3–S6† for reference Raman spectra of compounds.

In the present contribution, we show that a line-focused laser-based Raman spectrometer is well suited to obtain spectra simultaneously over a well-defined length (across a channel) to yield spatially resolved spectral data in real-time without movement of the laser or sample. The object plane is the cross-section of a microfluidic channel (Fig. 2). Each imaged row corresponds to a different position along the illuminated line, and therefore channel width. This approach overcomes a challenge in point-based Raman imaging, where each pixel needs to be read out in a consecutive manner. The system is applied to the characterisation of mixing induced by herringbone static mixers in microfluidic channels. We show that fast readout can be achieved without spectral overlap or shuttering using an sCMOS-based imaging detector. This approach enables imaging of processes such as mixing in microfluidic channels in real-time, allowing for the performance of static mixer designs to be evaluated rapidly for both miscible and immiscible solvent streams in the channel with the resonance-enhanced Raman scattering of isotopologues of a ruthenium(ii) polypyridyl complex enabling characterisation of the flow of chemically equivalent miscible solvent streams. Finally, we demonstrate that resonance enhancement can be used in combination with a fast chemical reaction, Scheme 1, to accurately determine the onset of effective mixing between two equivalent streams in the channel.

## 2 Experimental

### 2.1 Materials

All materials and solvents were obtained from commercial sources unless indicated otherwise. Solvents were of spectroscopic grade and used as received. [Ru([H_8_]bpy)_2_(bpt)](PF_6_) and [Ru([D_8_]bpy)_2_(bpt)](PF_6_), where bpy = 2,2′-bipyridine and bpt = 3,5-(bis)2′-pyridyl-1,2,4-triazolato anion (Scheme 1), were available from earlier studies.^36^

**Scheme 2:**
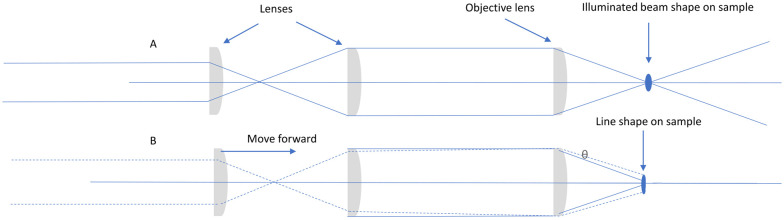
(A, top) Expansion using cylindrical lenses and (B, bottom) moving the first cylindrical lens out of focus to create a divergence or fan angle. Moving the cylindrical beam expansion lenses with respect to each other creates a tune-able fan angle, on the line expanded beam. This movement allows for tuning of the line size at the focal plane of the objective.

**Scheme 3:**
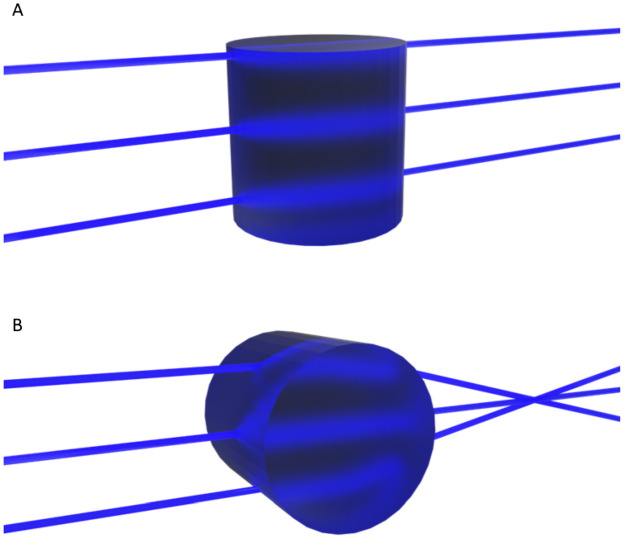
Ray-diagram through (A) the non-curved and (B) curved axes of a cylindrical lens. The beam is focused and subsequently diverges along one axis only.

Raman spectra were recorded at 473 nm (35 mW at sample, 04-01 series ‘blues’, Cobolt Lasers, Hubner Group, Sweden) either with point or line-shape illumination. A line was generated with a combination of cylindrical and planoconvex lenses (LJ1878L1 and LA1213, or LJ1695RM and LJ1703RM, Thorlabs, Scheme 2) held in rotation mounts. Specifically, the cylindrical lenses help to shape the laser-beam along one axis only (Scheme 3). Although other approaches for line-shaping are available,^21,37^ the present approach allowed for tuning of the line-shape, in particular to generate a near-collimated line oriented parallel to the spectrograph entrance slit. A detailed description of the procedure used for generating line-shaped illumination as well as its characterisation can be found in the ESI† and Fig. S1 and S2.

Signals were collected using the optical configuration in Fig. 2. A dichroic beamsplitter (Semrock Di02-R473-25) was used to bring the point or line source excitation beam, co-linear with the optical axis of the spectrometer, to the sample, through an Olympus 10× objective. It is of note that a higher magnification objective (and higher numerical aperture/NA) is often more efficient in the collection of Raman scattering. However, in the 180° backscattering arrangement used here, the objective lens is used both for the collection of light as well as illumination of the sample. In the present setup, the 10× objective is required to provide full overlap of the project illumination line with the channel width. In this arrangement, the laser provides sufficient photon flux that useful spectra can be recorded simultaneously over a few hundred microns and a reasonably flat intensity profile across the channel itself (see ESI†). This reduces the time needed to generate each image. Imaging of microfluidic channels in this way allows for real-time characterization of flow, with sub-second readout rates. Raman scattering was collected in 180° backscattering mode and passed through a Rayleigh rejection filter (longpass filter, Semrock LP02-473RE-25) before being focused with a 2.5 cm diameter (7.5 mm focal length) planoconvex lens into a Shamrock300i spectrograph. The spectrograph with two exit ports was equipped with both a Zyla 4.2-sCMOS camera and a Newton 460BV EMCCD camera (Andor Technology), as well as a 1200 l mm^−1^ grating blazed at 500 nm. Images were obtained using the sCMOS detector, unless indicated otherwise. Spectra were calibrated with the spectrum of cyclohexane (ASTM E1840-96 (Standards for Raman spectroscopy, 2002)). Beam shape was characterized using the Raman scattering from a 40-micron-thick polystyrene sheet (Fig. S2†). The uniform thickness results in differences in spectral intensity due only to the incident beam shape and intensity profile. Data was analyzed using SpectraGryph-12 and plotted using Python.

**Fig. 3:**
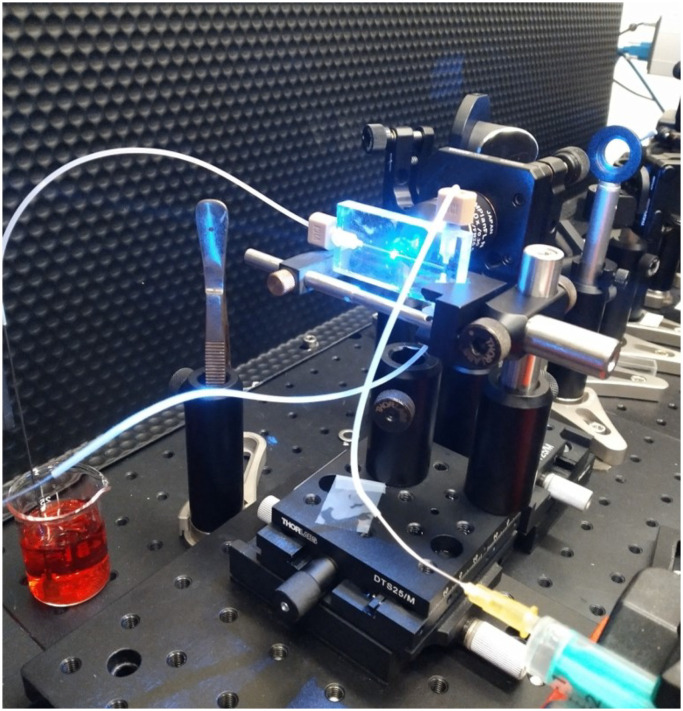
Quartz chip during *operando* analysis of the chemical reaction described in Scheme 1 with the line-focused 473 nm laser.

Microchannels were prepared with PDMS on glass slides as described elsewhere.^9^ Quartz microchannels with herringbone structures were fabricated by LightFab (Aachen, Germany) using groove-geometries optimized by Lynn and Dandy,^14^ and used as received. The quartz microfluidic chip used has two inlets merging with a T junction, followed by a channel section (10 mm in length) with smooth walls, which induces side-by-side flow when the solutions entering each of the inlets are of the same solvent. In the second part (20 mm in length) a herringbone groove structure is included in the bottom of the channel to form a static mixer. Each cycle consists of 2 half cycles with 6 grooves each. The half cycles differ by their groove apex offset location; one half cycle is right of center, other left of center. The channel width is 300 micron and channel height is 60 micron. The groove depth is 50 micron, the height over width channel aspect ratio is 0.2, the groove depth to channel height ratio is 0.8, the asymmetry index (apex location) is 0.62, *θ* (the groove intersection angle) is 90°, the grove width is 105 micron and the groove spacing is 50 micron as described earlier by Ianovska *et al.*^9^ After the mixing region, the channel is smooth again. HPLC plugs are used to connect PTFE tubes from the syringe pumps to the chip. The outlet is guided to a beaker for collection, Fig. 3. Images were obtained from quartz microchannels, unless indicated otherwise (see ESI† for a comparison of the Raman spectra of the various materials used, Fig. S7–S9†). For details of fabrication of the PDMS-based microfluidic chip see ESI.† Syringe pumps (New Era NE-1000, USA) were used to control solvent flow (Fig. 3).

A laser wavelength of 473 nm was selected for applications with poly(dimethylsiloxane) PDMS/glass microfluidic devices to avoid background emission arising from the chip material. However, the techniques described are not limited to specific excitation wavelengths. As an example, Raman spectral images generated at 785 nm (with a Cobolt 08-NLDM-ESP 400 mW laser) are shown in the ESI,† Fig. S10.

### 2.2 Imaging interface between immiscible solvents

**Fig. 4:**
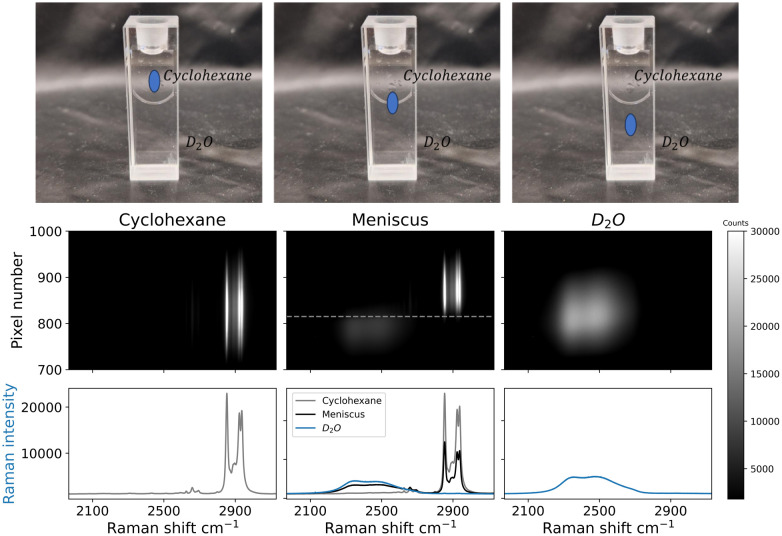
Raman spectral images recorded at 473 nm using an sCMOS detector. Bands are cyclohexane (left image, 2852 cm^−1^, 2923 cm^−1^ and 2938 cm^−1^), D_2_O (right image, 2500 cm^−1^), and the interface between the solvents (middle image) with Raman shift of both solvents, at different heights. The spatial separation is on the *y* axis, where each pixel corresponds to approx. 4 micron, with the magnification used. The spectra shown below each image are the sum of the whole or part of the image. (Photo) Analysis locations below, on, and above the meniscus.

Differences along the sample plane in the imaging approach were characterized by analysis of a solvent meniscus. An image obtained from two immiscible solvents, water and cyclohexane, in a cuvette shows a meniscus between these liquids (Fig. 4). Spatial separation of Raman scattering originating from separated solvent layers is apparent. The meniscus between two liquids depends on the contact area between them, where a longer pathlength yields a higher meniscus.^11,38^ Therefore, solvents were layered in a 1 mm pathlength cuvette to minimise the height of the meniscus. Deuterated water (D_2_O) was used to facilitate comparison of the solvent bands (the band of D_2_O is closer to cyclohexane than that of water due to the isotope shift of the Raman band). Spectral images were collected from different positions in the cuvette by pointing the line illumination below, at, and above the meniscus. As such, spectra of D_2_O and cyclohexane are collected separately, as well as both solvents simultaneously from measurement of the meniscus. The distance between two rows imaged on the detector is proportional to the distance between the respective points on the illuminated object. In the center of the image, contributions from Raman scattering originating from both liquids is observed. The rows of pixels where signals of both solvents overlap corresponds well to the meniscus height (105 micron).^11,38^ Additional contributions to spectral overlap are due to deviation in the position of the cell from perpendicularity to the optical axis, and differences in the refractive indices of the two solvents (cyclohexane – 1.42 *vs.* D_2_O – 1.328). Despite these physical sources of overlap and the meniscus itself, the two phases are essentially separate in the image.

## 3 Results and discussion

Raman spectroscopy is inherently confocal, meaning that a signal comes from a relatively well-defined volume in space. Typically, lasers used for Raman spectroscopy have a circular beam cross-section, or beam profile. The result of focusing this circular beam with an objective lens is a spot size having a diameter that corresponds closely to the width of the detector entrance slit (10–50 microns), Fig. 2. The Raman signal arises from this illuminated spot. This optical approach maximises the flux of Raman photons reaching a minimum number of pixels on the CCD detector array. Interference by shot- and readout-noise, as well as stray light, is then reduced, since only a narrow strip of the CCD needs to be read out to obtain the spectrum from the illuminated spot.

### 3.1 Imaging of a PDMS microchannel with a CCD camera

**Fig. 5:**
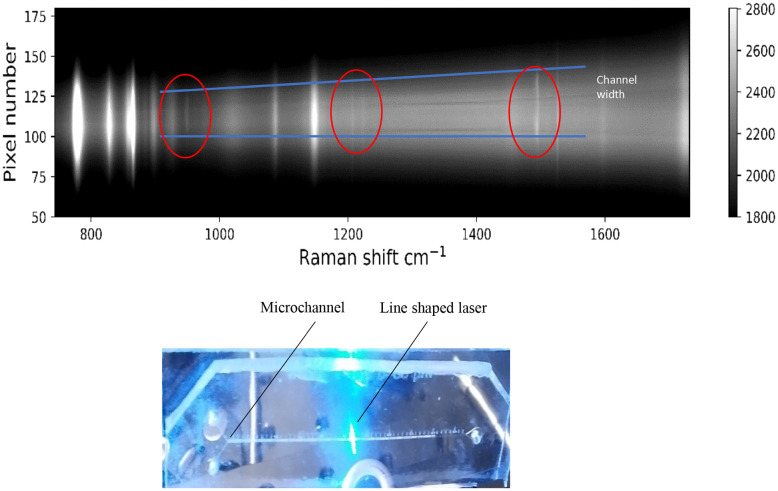
(Top) Spectral image recorded at 473 nm with a CCD detector in imaging mode showing the cross-section of a microchannel (outlined in blue) filled with acetonitrile (bands encircled in red). Raman shift is on the *x*-axis, and relative spatial separation (channel width) on the *y*-axis. (Bottom) Photograph of 473 nm laser (blue) line source illuminating perpendicularly to a horizontal PDMS channel direction. In order to prevent streaking caused by the readout of the CCD detector, the spectrograph was shuttered entirely during readout of the chip to obtain the image. For reference Raman spectra of PDMS and acetonitrile see Fig. S11.†

The line-focus approach is demonstrated using a PDMS-on-glass microfluidic device of known dimensions (PDMS channel width of 300 microns and 100-micron depth) containing acetonitrile. The channel was imaged spectrally (Fig. S11† for spectra of PDMS and acetonitrile). The known width was used to characterize the optical system with regard to magnification at the detector. The spectral image, Fig. 5, was recorded on a CCD detector with the line-expanded excitation laser (473 nm) aligned across the width of the channel. The spectrometer was shuttered during readout of the CCD to avoid spectral cross-talk (smearing, Fig. S13†).

Raman bands associated with PDMS span the entire illuminated range, and thus extend beyond the width of the channel. The bands due to scattering from acetonitrile inside the channel, circled in red, only appear in the central 24 pixels, which corresponds closely to the channel width of 300 micron (24 pixels × 13 micron per pixel), at zero magnification. The lines diverge vertically at the sides of the image, because of Petzval field curvature (see ESI† for details). Raman scattering provides sharp bands in the recorded spectrum; therefore, bands of the solvent are easily distinguished from the bands arising from the PDMS chip material.

Detectors based on cooled CCD arrays are well suited to low light conditions, such as Raman spectroscopy. However, a challenge faced by CCD technology in spectral imaging, (*i.e.* the simultaneous acquisition of spectra over a significant length scale such as the width of a microfluidic channel (50 to 400 microns)), is spectral cross-talk. This cross-talk is due to the overlap of signals imaged from different points in the channel onto the same pixel as a result of the vertical readout method employed by CCDs.^39^ Using a physical shutter during readout of the detector can avoid this issue, but at the cost of achievable imaging frequency. An example of spectral smearing and the readout sequence for a CCD is shown in the ESI† (Fig. S13 and S14).

One approach taken to circumvent this phenomenon is the custom built photodiode array reported by Nitta *et al.*,^7^ where the photodiodes (PDs) are read out individually. Each PD in the array defines a pixel in the image. This method of readout is analogous to the readout sequence used in a complementary mixed oxide semiconductor (CMOS) based array camera (US patent US5841126A), where each pixel on the chip is also read out individually.^40^

The chip layout of a CMOS camera facilitates high readout rates (up to 100 times faster than CCDs), since each pixel is read out individually. Therefore, there is less need for a shutter, if any, and spectral cross-talk (smearing) is avoided. However, CMOS sensors typically show higher readout noise than cooled CCDs, and are mostly applied in high-photon-flux applications where fast readout and time-dependent measurements are required.^41,42^ Hence, CMOS-based sensors have received relatively little attention for Raman spectroscopic imaging, despite their lower cost compared to CCDs. However, the lower cost has resulted in the rapid replacement of CCDs,^43^ especially in applications such as consumer cameras, phones and also microelectromechanical systems (MEMS).^44^ The rapidly increasing performance of CMOS chips^45–47^ has resulted in the advent of scientific CMOS (sCMOS), in which the ‘scientific’ prefix denotes the newest generation of CMOS detectors, with higher signal-to-noise ratios and rapid frame rates. This development means they should be considered in areas currently dominated by CCDs.^39^ For imaging purposes in particular, sCMOS detection technology holds advantages over CCD detectors. Therefore, sCMOS detection is used in the recording of Raman spectral images described further here.

### 3.2 Mixing of immiscible liquids

**Fig. 6:**
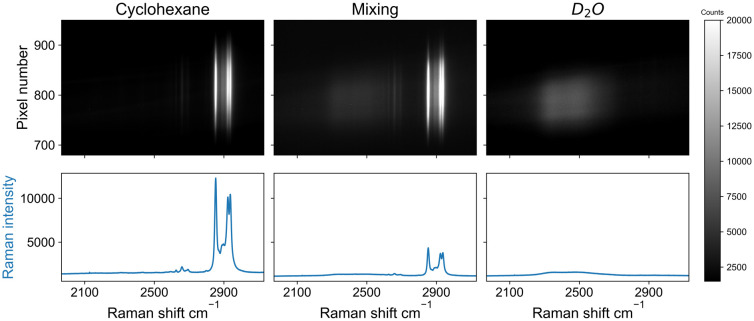
Raman spectral images of a quartz microchannel recorded at 473 nm with an sCMOS detector. The channel contains cyclohexane and D_2_O, fed into the chip at 100 μL min^−1^ each. Images show slugs of (left) cyclohexane, (right) D_2_O and (middle) both solvents mixed. Left and right images are recorded in the smooth-walled section of the channel and the middle image is recorded in the mixing domain of the chip. Spectral features of both solvents are visible in the domain of the chip that contains herringbone grooves. It is expected here that the slug flow is broken up by the mixing and forms an emulsion. The optical axis and the long axis of the laser line were orthogonal to the channel length. Bands are D_2_O (2500 cm^−1^) and cyclohexane (2852 cm^−1^, 2923 cm^−1^, 2938 cm^−1^).

The flow of immiscible solvents (water and cyclohexane) in a quartz microfluidic chip was studied by line-focused Raman spectroscopy (Fig. 6). The first part of the channel contains a smooth-walled (featureless) section followed by a section with herringbone grooves that act as a static micromixer (Fig. 1). The length of the laser line at the focal point was adjusted to be greater than the width of the channels to ensure that the intensity was approximately uniform over the width of the channel (*vide supra*). A slug-flow pattern is observed in the smooth-walled region of the channel. Time-resolved Raman spectral images clearly show alternating slugs of each phase passing through the confocal volume, see ESI,† Video S1 for Raman images of slugs passing in real-time. The observation of slug flow in micro-flow is as expected.^19^ A spectral image obtained with the line illumination parallel to the flow direction shows the size of a slug, Fig. S12.† The middle image, measured over the mixing domain, shows the presence of both D_2_O and cyclohexane over the entire width of the channel but each with lower intensity. The presence of both bands across the channel indicates physical mixing of the two streams. As the liquids are immiscible, perturbed laminar flow in the form of slug flow is expected, (*e.g.*, an emulsion) with several bubbles of each solvent in the depth of the confocal volume. The presence of spectral features for both components across the width of the channel confirms a disturbance of flow by the grooves. However, conclusions drawn from analysis of mixtures of immiscible liquids cannot be extended to more commonly used mixtures such as water/water which show side-by-side flow.

### 3.3 Laminar flow and mixing of miscible solvents

Mixing flows of identical liquid streams (*i.e.* water/water), presents a challenge in characterisation due to their Raman spectra being identical. Therefore, for the investigation of side-by-side flow and mixing of miscible solvent streams with identical Raman spectra, an alternative approach is needed. The enhancement of Raman scattering observed under resonant conditions (resonance Raman spectroscopy) allows for observation of solutes at less than millimolar concentrations (*vide supra*).^31^ Hence, the flow and mixing of two chemically identical streams of solvent can be studied using two solutions, each containing a different isotopologue of a compound that shows resonance enhancement. The Raman spectra of the photochemically and thermally stable complexes [Ru([H_8_]bpy)_2_(bpt)]^+^ and [Ru([D_8_]bpy)_2_(bpt)]^+^ in water at low (0.1 mM) concentrations show intense narrow bands in the 1400–1600 cm^−1^ region. Two aqueous solutions, each containing one of the two isotopologues, enter *via* separate inlets into the quartz microchannel at equal rates to achieve stable side-by-side flow. Although the solutions are physically and chemically equivalent, deuteration allows for distinguishing between the resonantly enhanced Raman scattering of the complexes dissolved in each stream (Fig. S6†). This approach has the advantage of avoiding effects due to differences in the physical and chemical properties of the solvent, *e.g.*, osmotic pressure, phase separation, or reactivity between solvents and solutes.

**Fig. 7:**
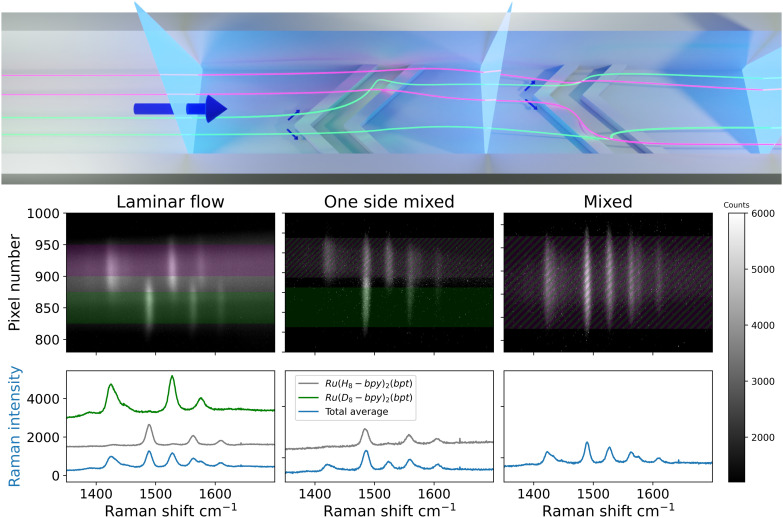
(Top) Locations analysed (vertical blue sheets) in the microchannel. The two liquid streams (represented as purple and green) enter side-by-side. The off-center apex of the grooves mixes one side first, and upon re-centering the other side is mixed as well. (Bottom) Raman spectral images of a quartz microchannel recorded at 473 nm with a sCMOS detector, (left) in the region of laminar flow, (middle) partial mixed flow in a microchannel and (right) fully mixed flow. The optical axis and the long axis of the laser line were orthogonal to channel length. The Raman bands for (purple) [Ru([H_8_]bpy)_2_(bpt)]^+^ (1490 cm^−1^, 1563 cm^−1^, 1610 cm^−1^) and (green) [Ru([D_8_]bpy)_2_(bpt)]^+^ (1425 cm^−1^, 1527 cm^−1^, 1575 cm^−1^) are due to resonance enhanced Raman scattering from the 2,2′-bipyridine ligands.

The extent of mixing between the solvent streams was determined at several positions along the length of the channel, Fig. 7. The first image shows side-by-side flow, with some spatial overlap of the Raman bands of the complexes in the spectra at the center of the image. The overlap is stable over the smooth-walled section and is due to imperfect alignment of the interface between the solvent streams and the optical axis of the spectrometer. This highlights that the depth of the confocal volume along the spectrometer axis is greater than the depth of the channel (*vide infra*). The second image shows contributions from both streams in one side of the channel only (top side). Earlier studies have shown that solvent streams begin to tumble over one another at the point within the microchannel at which the grooves begin; first on one side, then the other side of the channel.^10,14^ This folding happens in an asymmetric manner, Fig. 1, caused by the alternating apex location of the herringbone grooves. The Raman image recorded at this point in the channel shows this first folding of the bottom side of the channel onto the top side, manifested in overlap of the spectra of the two isotopologues in the top half of the channel.^9,10,14^ The third image shows that Raman bands of both isotopologues are observed over the entire width of the channel suggesting complete mixing of the two streams. It is, however, not possible to distinguish between two separate, folded flows or fully mixed flow based on this image. The observation of bands from different analytes on top of each other can also be caused, however unlikely, by streams flowing side by side but rotated by 90°; the limited *z*-confocality means Raman scattering is collected from the entire depth of the channel. Ultimately this approach is equivalent, with regard to the information obtained, to earlier studies using fluorescence microscopy (*vide supra*). Therefore, another approach to determine completeness of mixing, that circumvents the need for greater depth resolution, is required.

### 3.4 Chaotic mixing of two equivalent solvent streams using in-line reaction monitoring

An alternative approach to studying the extent of mixing is to use a fast chemical reaction occurring in the channel, with the reagents entering *via* separate liquid streams. Reagent molecules need to be in sufficient proximity to collide for the reaction to occur. Therefore, the extent of reaction is a measure of extent of mixing.

The reaction between the colourless iron(ii) sulphate (Fe(ii)SO_4_) and 2,2′-bipyridine (bipy) is fast, essentially diffusion controlled, and yields the bright red complex [Fe(ii)(bipy)_3_]^2+^, Scheme 1. The product has a characteristic visible absorption spectrum (Fig. S16†). This reaction was chosen to study mixing in the channel as the visible absorption bands of the complex [Fe(ii)(bipy)_3_]^2+^ are resonant with the laser used (473 nm). The reagents were introduced into the channel separately in each of the two inlet streams. One aqueous stream contained 1.2 mM Fe(ii)(SO_4_) in phosphate buffer (pH 7.4), the other, 3.6 mM bipy in phosphate buffer (pH 7.4). The concentrations of these reagents were too low to give significant non-resonant Raman scattering (see Fig. S3 and S4† for reference spectra of the compounds). The product [Fe(ii)(bipy)_3_]^2+^ shows well-defined Raman bands at this concentration due to resonance enhancement (Fig. S5†). The progression of this chemical reaction is manifested in the Raman bands observed.

Raman images of a quartz microchannel with water show the cross-section illuminated by the line-focused laser. The bands of quartz (<1200 cm^−1^) appear in the center pixels of the image, *ca.* 300 pixels in height. Those of water (1600 cm^−1^) appear in the center also, but over a narrower range, *ca.* 250 pixels, showing that the illumination exceeds the width of the channel, Fig. S15.†

**Fig. 8:**
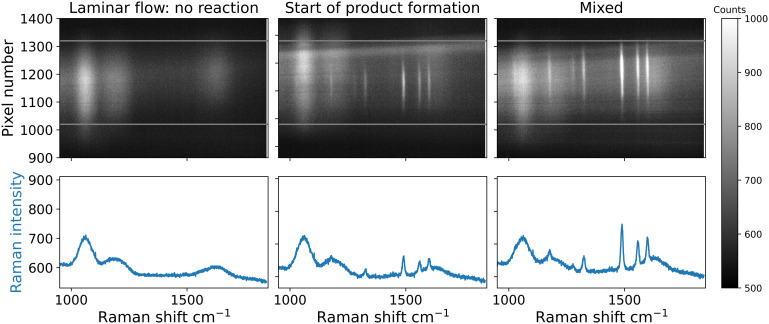
Raman spectral images taken at locations along the length of the channel, see Fig. 7. From left to right, the first point is laminar flow, the second indicates the start of product formation centered around the first ridge in the microchannel, and the final point is the maximum amount of product observed at the end of the microchannel. Bands centered around 1500 cm^−1^ associated with the deep red complex [Fe(ii)(bipy)_3_]^2+^ arise from the middle of the channel to the entire width of the channel within a few micrometers. Maximum signal was observed at 30 mm distance into the channel, right after the grooves. After the grooves end, further changes are not observed. Fe(ii)(SO_4_) (1.2 mM) was dissolved in phosphate buffer (pH = 7.4) and fed into the first inlet, and 2,2′-bipyridine (3.6 mM, phosphate buffer) was fed into the second inlet, with a flow rate of 100 μl min^−1^ each.

Side-by-side flow of the two streams in the channel prevents significant mixing of the solutions. Hence, the reagents in each solution cannot be seen in the Raman spectra obtained in the region where stable side-by-side flow is obtained and only the water and quartz bands are observed (first 10 mm, Fig. 8, first image). Specifically, the flow rate (100 μL min^−1^) was too high to allow for diffusion to mix the liquid streams within their residence time in the microchannel (67 ms), *i.e.* the Peclet number (ratio of convective to diffusive mass transport) for this device is 3 × 10^4^, so mixing is predominantly reliant on convection.^9^

Indeed, when convection is applied (when the grooves start) in the channel, the Raman spectrum of the product formed upon mixing is readily observed, Fig. 8 (middle and last image). As observed earlier (*vide supra*) the solvent streams mix first on one side of the channel, to partly form the product. In subsequent periods of the herringbone grooves, the product is formed over the entire width of the channel, which is in agreement with observations with the ruthenium(ii) complexes above (Fig. 7) and reports on fluid behaviour inside the channel.^9,10,14^

Notably, at a point just before the solvent streams reach the herringbone pattern (static mixer), the perturbation caused by the ridges provides for mixing due to disturbed laminar flow. Here, the Raman bands of the product are observed at the apex of the first groove. After the point at which the flow has passed one cycle of herringbone grooves, the bands of the product are observed over the entire width of the channel. The observation of essentially instantaneous formation of [Fe(ii)(bipy)_3_]^2+^ is expected due to the high reaction rate. In the region after the mixing structures, further changes in the spectra are not observed.

## 4 Conclusion

Raman spectroscopy provides a spectral fingerprint enabling label-free discrimination of immiscible solvent streams. For side-by-side flow of identical solvent streams, isotope labelling of solutes together with resonance enhancement allows for the flow of each solvent stream to be observed in a manner analogous to fluorescent probes.^9^ Although greater spectral resolution is achieved with resonance Raman spectroscopy, both approaches are limited by *z*-confocality (depth resolution in the channel). This limitation is overcome by using resonance enhancement together with a fast chemical reaction to determine the extent of effective mixing of solutes between streams as they encounter groove structures. The observation of Raman bands of the product of the reaction indicates extent of mixing.

A useful aspect of flow chemistry is correlation between the extent of reaction and the distance travelled by the solution in the channel/tube. Hence, under conditions of constant flow rate, the extent of the reaction at any point in the channel does not change over time. Spectra can be recorded with a temporal precision that is limited only by the spatial resolution of the spectral acquisition. With the line-focus method, variations in reaction extent across the channel width can be observed as well.

From a practical perspective, although the sensitivity of sCMOS detectors is less than CCD detectors, they have the benefit of not needing to be shuttered during readout. This is because cross-talk/smearing has less impact due to the manner by which the sCMOS chip is read out (each pixel individually). This readout advantage makes sCMOS useful for imaging applications such as the one described here.

The line-focus approach can be employed in steps along the length of the channel as distance along the channel is proportional to reaction time. Therefore, longer acquisition times can be achieved without losing temporal resolution, thereby overcoming the higher readout noise of sCMOS sensors. The approach taken here can be used as well to study fast (chemical) reactions with correction for the time taken for full mixing of solvent streams. We anticipate that the combination of spatial resolution achievable with sCMOS detectors and line focusing can together open opportunities for applications involving rapid spectral imaging.

## Author contributions

The research was conceived by WJNK, EV, and WRB. The data was obtained and analyzed by WJNK. The paper was written with contributions from all authors.

## Conflicts of interest

There are no conflicts of interest to declare.

## Supplementary Material

LC-024-D4LC00115J-s001

LC-024-D4LC00115J-s002
