# Supplementary material for: In-line Raman imaging of mixing by herringbone grooves in microfluidic channels
Source: Lab Chip. 2024 Jun 17;24(14):3498–507. doi: 10.1039/d4lc00115j (PMC11235414; doi:10.1039/d4lc00115j)
Supplement: LC-024-D4LC00115J-s001 [file LC-024-D4LC00115J-s001.pdf]

Electronic Supplementary Material (ESI) for Lab on a Chip.
This journal is © The Royal Society of Chemistry 2024

In-line Raman Imaging of Mixing by Herringbone Grooves in Microfluidic Channels

W.J. Niels Klement,[a,b] Elia Savino,[a] Wesley R. Browne*,[a] and Elisabeth Verpoorte,*,[b]

Supporting information

Shaping of the excitation beam

The shape of the confocal volume can be altered by shaping the incident beam, for example, by expanding the beam diameter (using a beam expander) to allow for increased z-confocality.[21,37] In the present study, we shape the incident beam to produce a line at the focal point of the object lens. This line is parallel with the spectrometer entrance slit. The change in the shape of the output beam of the laser from a circle to a line is achieved using two cylindrical lenses in series (Scheme 3). The desired beam properties can also be achieved with a single optical element, *e.g.*, a Powell lens, or with a line generated directly by the laser itself (as in the example at 785 nm shown in Figure S10, which uses a special laser that produces a beam with a fan angle). However, these approaches are limited by the range of dimensions that the beam can have (vide infra). Using two optical elements to shape the beam increases adaptability of the setup, by allowing finer control of the final beam shape by changing the distance between the lenses, Scheme 2. The control over beam shape allows analysis of various channel dimensions using the same setup. The divergence used in most experiments was 5 degrees, which was found to yield an illuminated line of approximately 500 micron in height along the axis of the slit of the spectrometer. This illumination size corresponds well with the dimensions of the microchannel used in the experiments described below (300-400 micron). A key aspect of the line-shape illumination is that the orientation of the long axis of the excitation beam is parallel to the entrance slit of the spectrograph. If the illumination is not parallel with the spectrograph slit, the image will be clipped as in the case of the 90° (orthogonal) line orientation in Figure S1. The orthogonal orientation effectively crops Raman scattering from either side of the confocal volume. During alignment, the spectrometer slit width was set to 2500 micron, with the grating at zero order (hence acting as a mirror), to collect light from the entire volume illuminated by the beam, to facilitate beam alignment.

The images shown in Figure S1 are of a polystyrene sheet with a uniform thickness of 40 micron, before and after placing the beam shaping lenses in the path of the laser. Several orientations of the beam were obtained by rotating the cylindrical lenses from perpendicular to parallel with respect to the slit direction. In the parallel orientation, the spatial information provided by the Raman scattering along the illuminated line is retained in the image. By dispersing the spectra across the sensor, differences in analyte composition along the illuminated line can be observed.

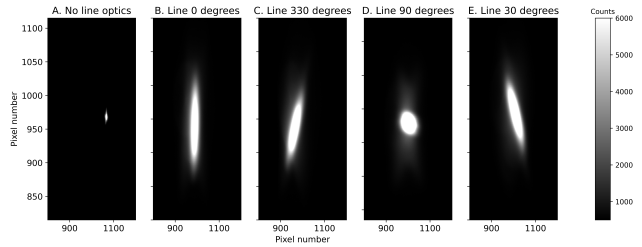

Figure S1 Beam shape, imaged on a sCMOS detector with the grating of the spectrograph set to zero order, (A) without shaping of the laser beam, (B) with shaping of the beam parallel to the slit, and (C-E) with beam shaping at various angles with respect to the spectrograph entrance slit. At 90 degrees (D), clipping of the beam by the spectrograph entrance slit is apparent. Part of the imaged line is blocked from entering the spectrometer and is manifested as a spot instead. When parallel with the slit, the line is approximately 500 micron in length.

The intensity profile of the line-expanded laser beam was evaluated using the same 40-micron-thick polystyrene film. With a constant sample thickness, the resulting intensity profile is proportional primarily to the intensity of the laser over the length of the focused line (Figure S2). The graph on the right side of the image denotes the intensity profile, averaged over each row of the detector. The observed Gaussian shape is to be expected from an optically expanded laser beam.[20] This intensity profile should be accounted for when quantitative measurements are desired using absolute intensity. However, an advantage of Raman imaging of solutes in solvent streams is that the solvent Raman bands can be used for intensity normalisation (internal reference). Variation in intensity can be partially avoided using a Powell lens, albeit that the divergence of Powell lenses is generally (too) large for application on micron-sized objects. Alternatively, variations in intensities in a measurement can be minimised by using only a small section of the line-expanded beam, for example the center section where the intensity profile is approximately flat. This is an approach commonly used in super resolution imaging.[47] A lateral divergence of the illumination can be observed in the spectral image (Figure S2), *i.e.* the lines in the image are shorter on the left side and longer on the right side. This divergence arises from Petzval field curvature, due to the curved focal plane of the spectrograph at the detector. Imaged bands can vary in size when optical magnification is changed, or when the lens system in the spectrograph is changed. The pixel height of the bands observed needs to be calibrated to a sample of known dimensions

to determine spatial resolution in the spectral image.

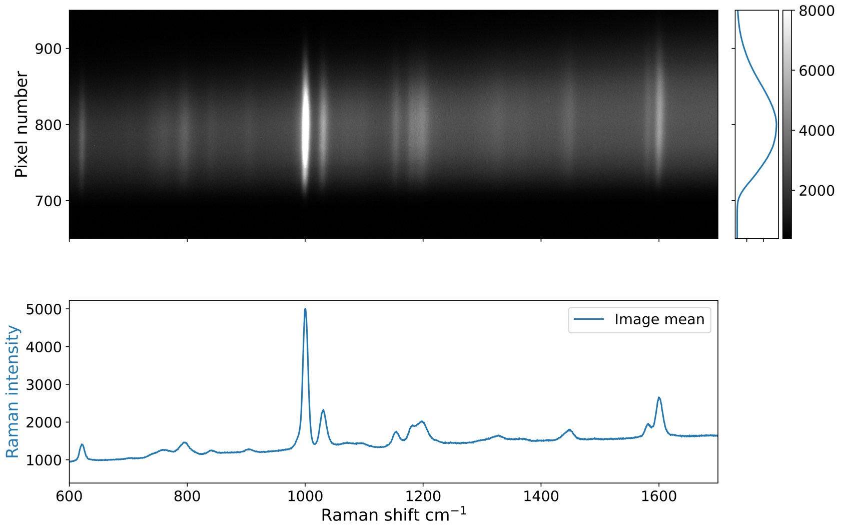

Figure S2 (Top) Raman spectral image of 40-micron-thick polystyrene calibration slide, (right) mean intensity, corresponding to intensity of laser along the line, (bottom) vertical mean of the image showing the spectrum of polystyrene.

Fabrication of PDMS on glass microfluidic devices

The PDMS on glass microchannels were fabricated using standard lift-off (soft) lithography methods. A 3D shape of the channel, the master, was drawn using CleWin32 software. This drawing was printed at ProArt BV, Groningen, The Netherlands. The two layers were, in turn, fabricated onto the glass base of the master using SU photoresist (Micro resist technology GmbH, Berlin, Germany) and a UV illumination.

The 3D structure was in PDMS was obtained by pouring liquid PDMS monomer containing 10 percent hardener (PDMS, Sylgard 184, Mavom BV, Alphen a/d Rijn, The Netherlands) onto the mold. The mold, including PDMS, was heated at 70C for 30 minutes, after which the PDMS was peeled of and cut into the desired shapes. In for the chips used here, one wafer produced 4 rectangular pieces containing a single microchannel, roughly of the same size as a microscopy slide (2.4x6 cm). The glass part did not require additional preparation. Standart microscopy slides were used (Knittel Glass, Glasbearbeitungs GmbH, Braunschweig, Germany). Glass and PDMS parts were attached together by putting the two in oxygen plasma for 30 seconds followed by lightly pressing together.

Reference spectra

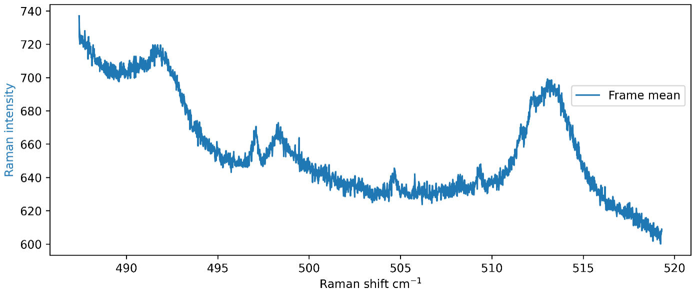

Figure S3 Raman spectrum of 2,2-bipyridine in water (3 mM) recorded in a 1 cm pathlength quartz cuvette. λ_{exc} 473 nm.

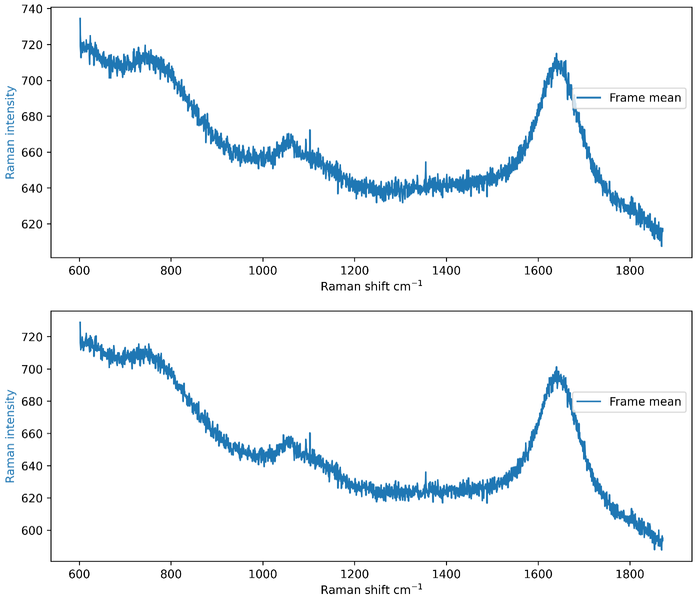

Figure S4 (top) Raman spectrum of iron(II) sulfate in water (1 mM). The concentration of iron(II) sulfate is below the limit of detection and only Raman bands from quartz and water are observed, (bottom) Raman spectrum of water. Spectra were recorded in a 1 cm pathlength quartz cuvette. λ_{exc} 473 nm.

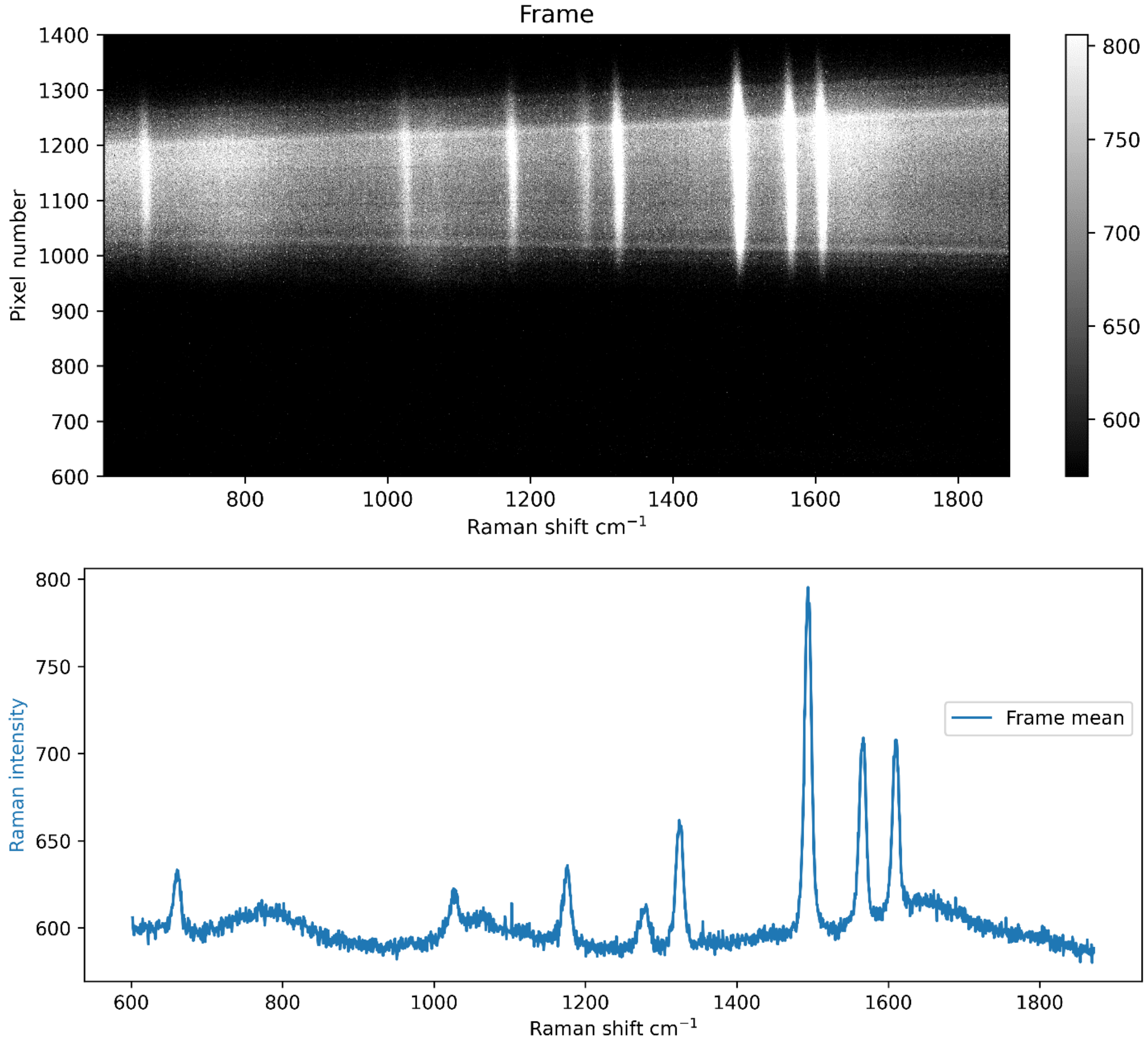

Figure S5 (Top) Raman spectral image of $[Fe(bipy)_3]^{2+}$ in water (1 mM) (Bottom) Raman spectrum of $[Fe(bipy)_3]^{2+}$. Spectra were obtained by binning data points from the frame vertically. λ_{exc} 473 nm.

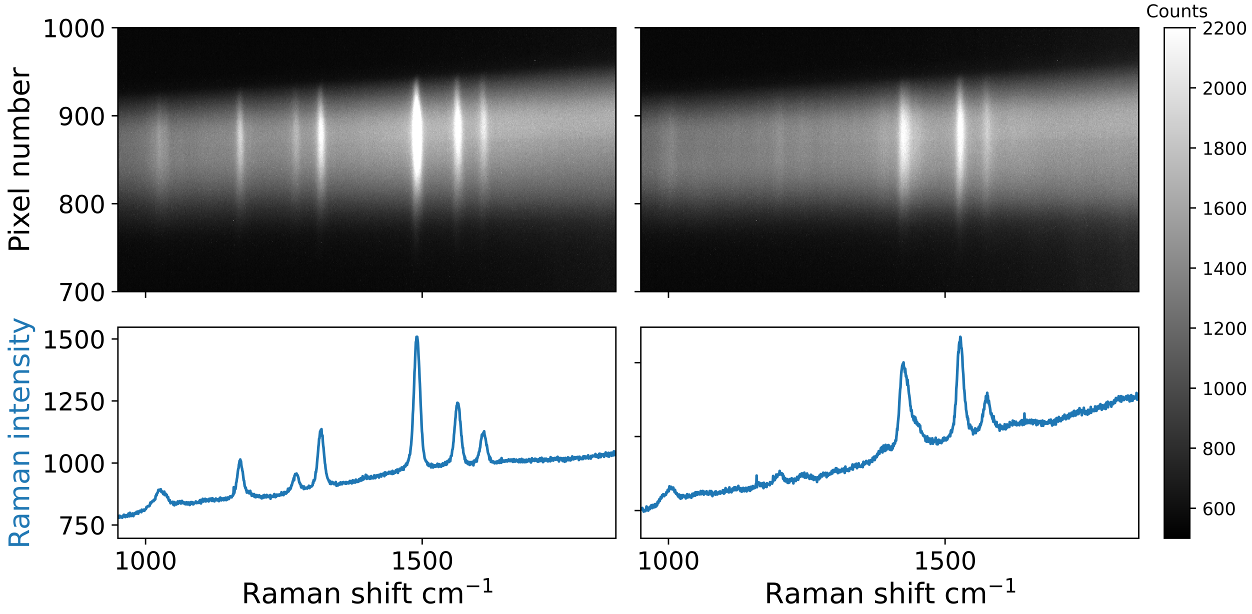

Figure S6 Raman spectra and spectral images of left: $[Ru(H_8{-}bipy)_2(bpt)]^{2+}$ and right: $[Ru(D_8{-}bipy)_2(bpt)]^{2+}$ in water (1 mM), recorded at 473 nm.

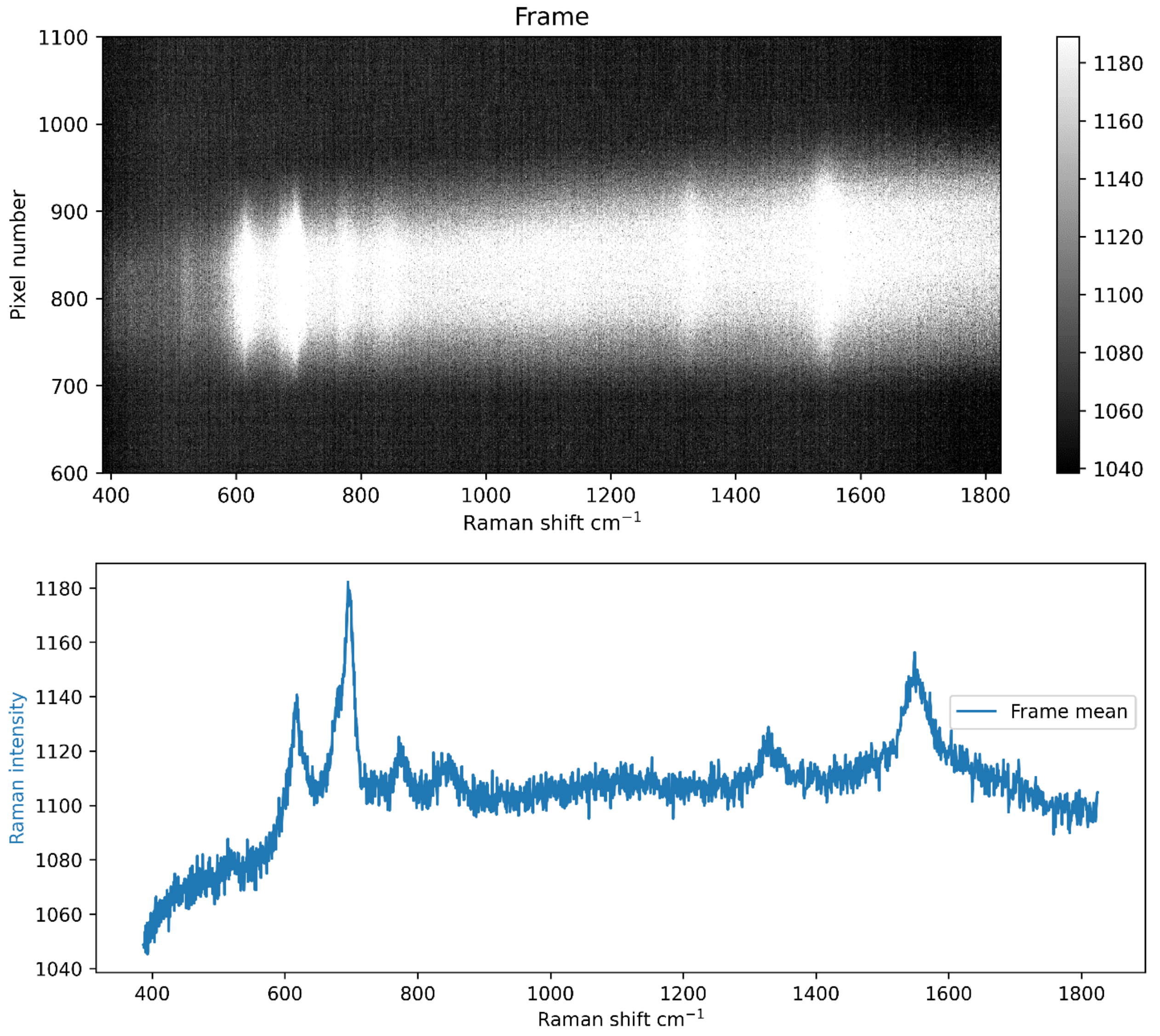

Figure S7 (Top) Raman spectral image of PDMS (Bottom) Raman spectrum of PDMS. Spectra were obtained by binning data points from the frame vertically. λ_{exc} 473 nm.

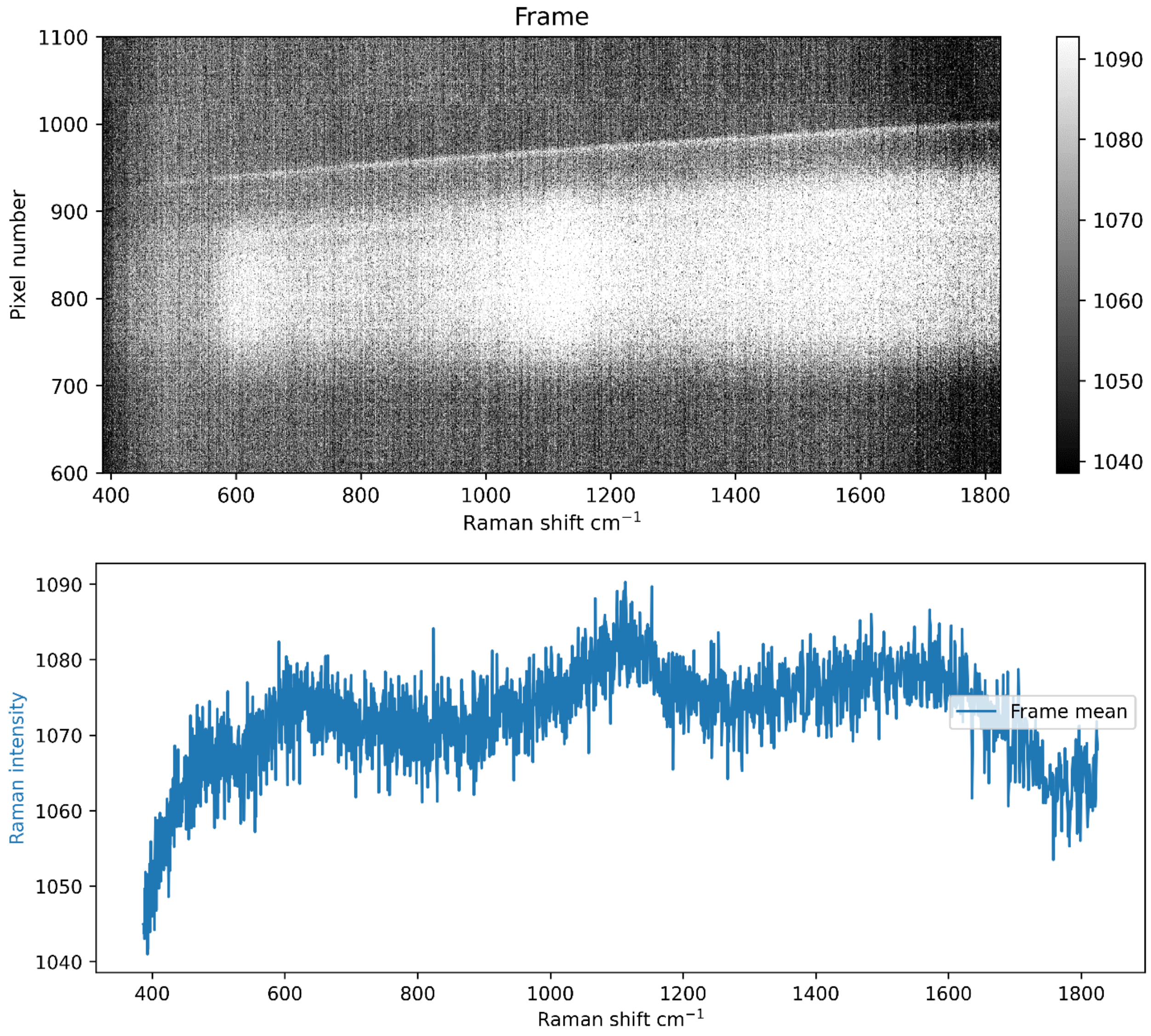

Figure S8 (Top) Raman spectral image of Glass (Bottom) Raman spectrum of Glass. Spectra were obtained by binning datapoints from the frame vertically. λ_{exc} 473 nm.

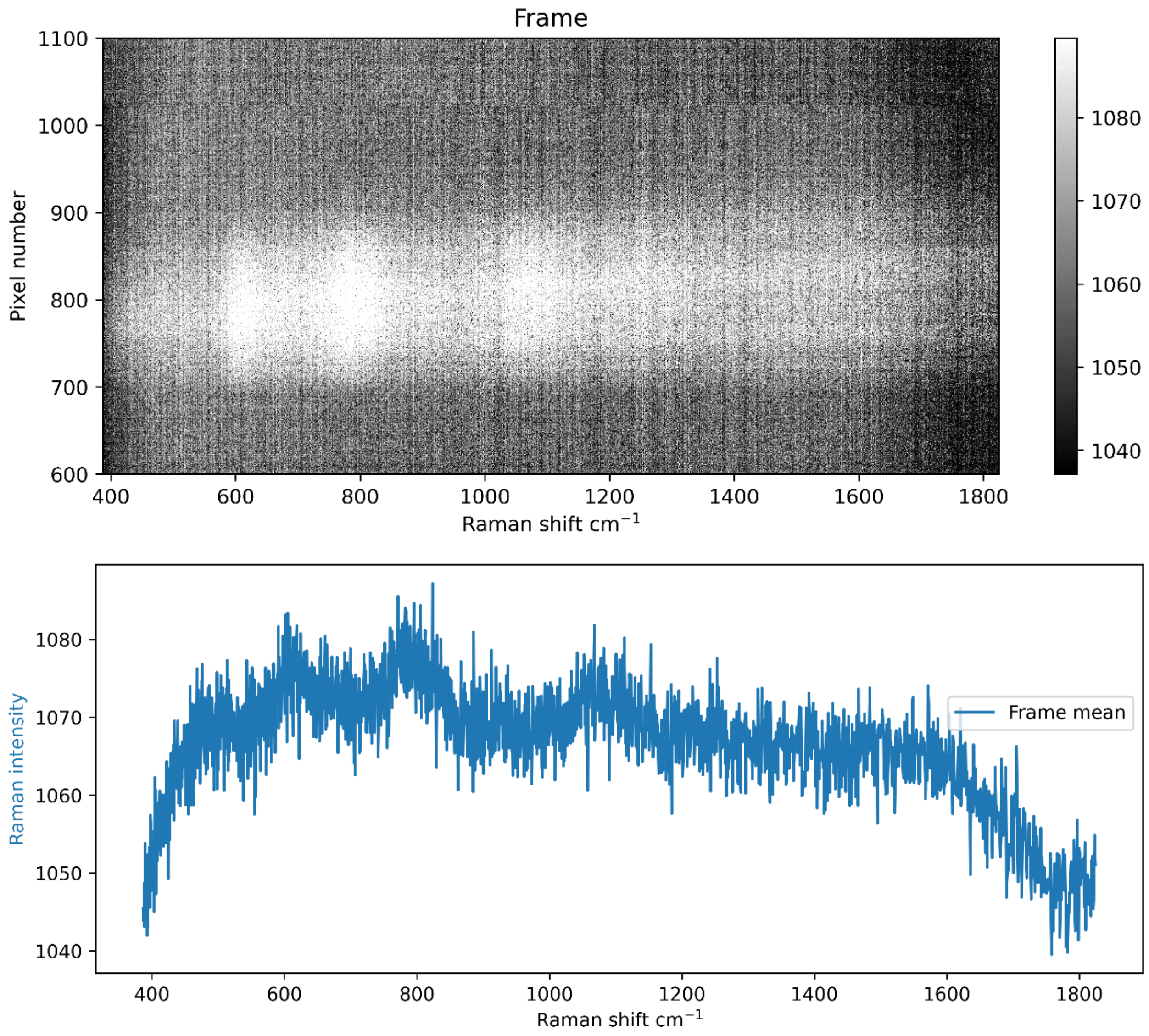

Figure S9 (Top) Raman spectral image of Quartz (Bottom) Raman spectrum of Quartz. Spectra were obtained by binning datapoints from the frame vertically. λ_{exc} 473 nm.

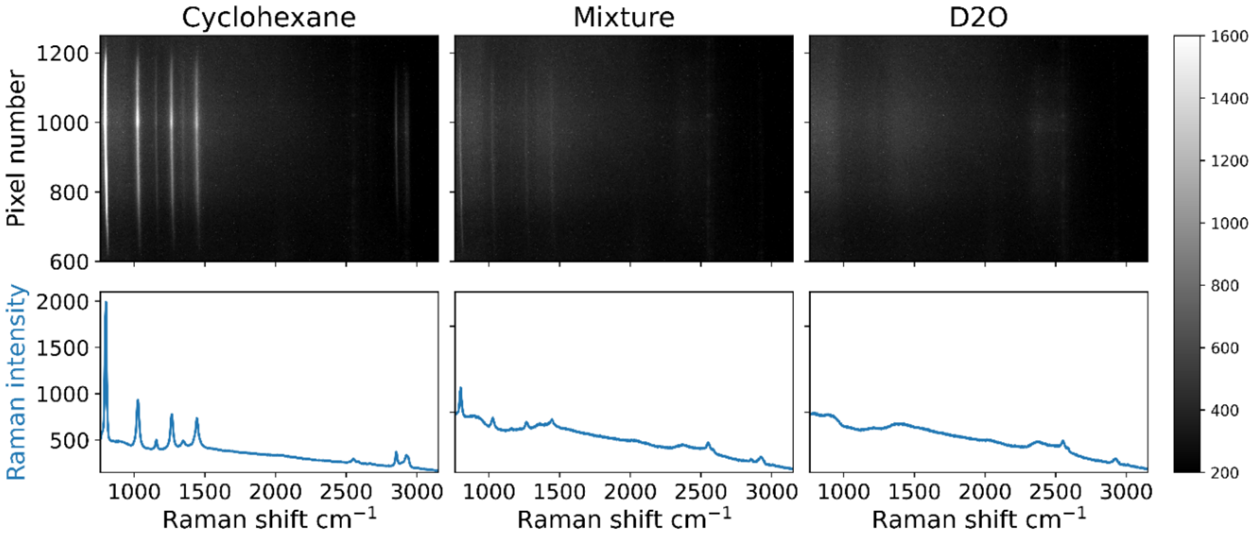

Figure S10 Images recorded at λ_{exc} 785 nm with a laser that has a line shaped output and an sCMOS detector in imaging mode. Bands are cyclohexane (sharp bands, 800-1500 and 2800 cm^{-1}) and D_2O (broad band, 2500 cm^{-1}). The middle image shows a mixed emulsion of the two liquids, obtained with vigorous stirring in the cuvette.

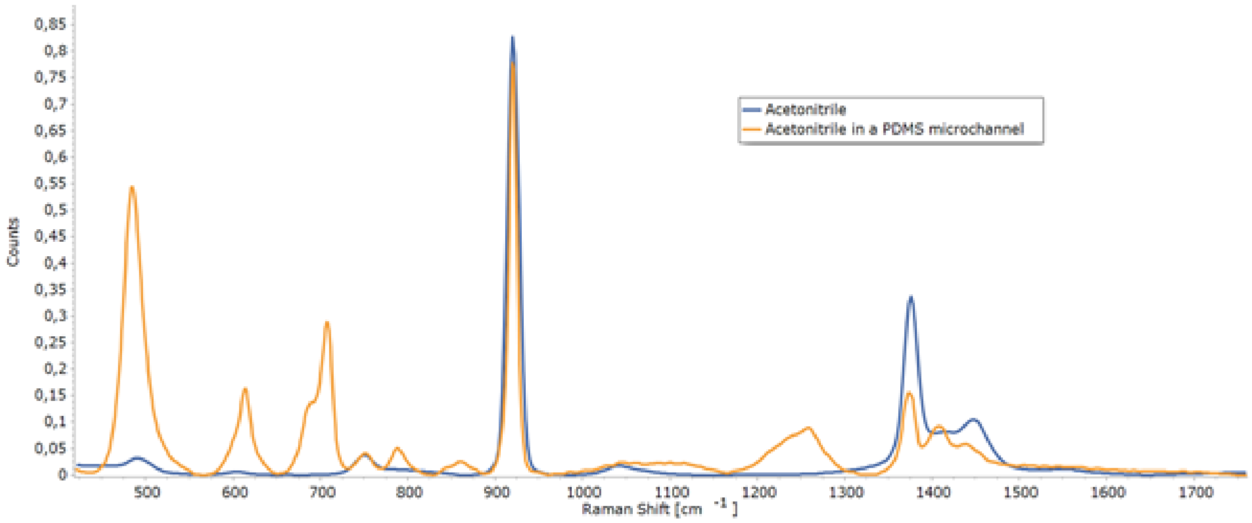

Figure S11 Raman spectra (785 nm) of (blue) acetonitrile and (yellow) acetonitrile in a PDMS microchannel.

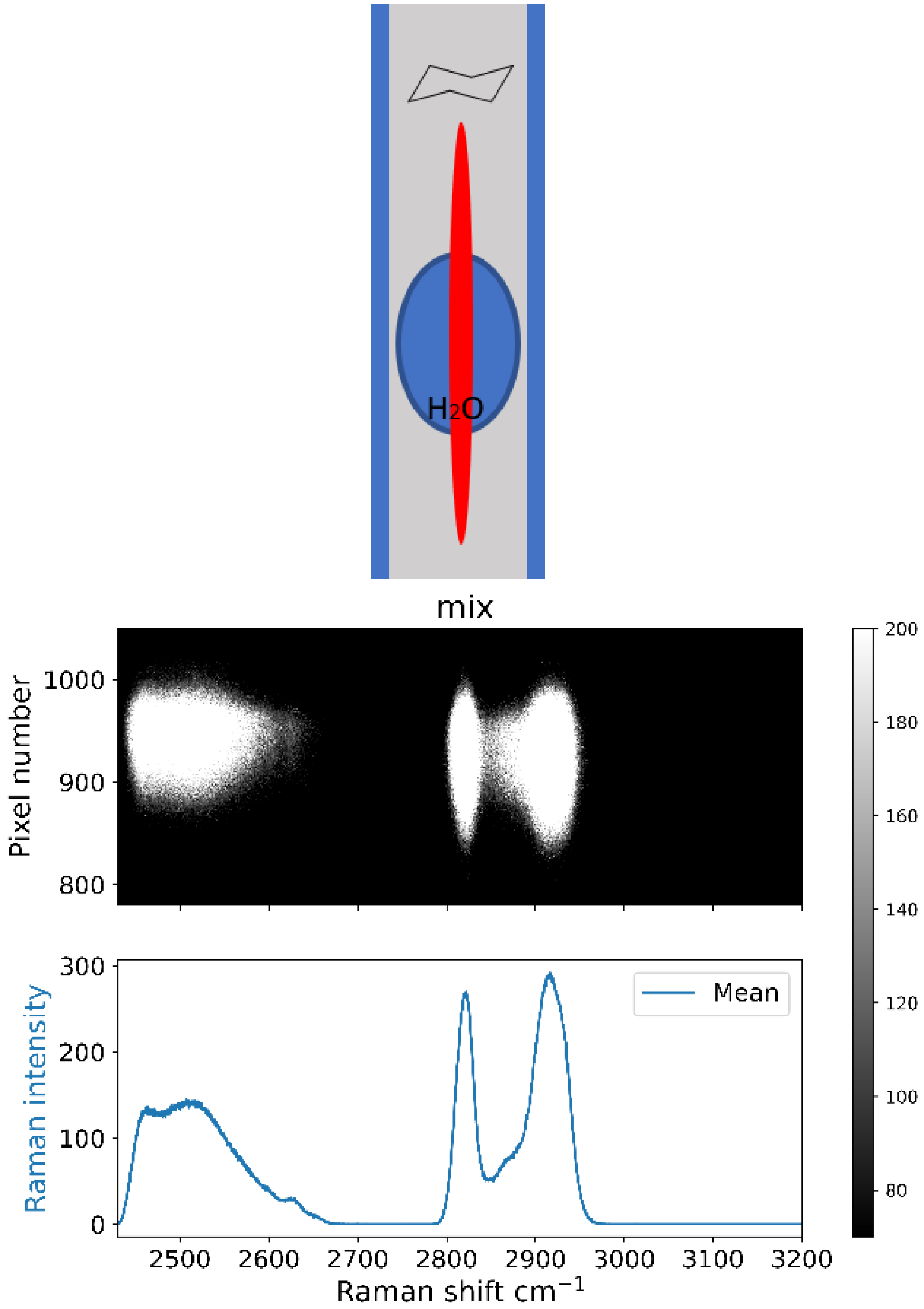

Figure S12 (top) Slug of D_2O in a cyclohexane filled microchannel,(middle) Spectral image of liquids in the mixed region, 100 μl/min. Images were recorded λ_{exc} 473 nm with an sCMOS detector in imaging mode, parallel to the direction of flow. Bands are D_2O (2500 cm^{-1}) and cyclohexane (2820 cm^{-1}, 2910 cm^{-1}). (bottom) spectrum obtained following full vertical binning

Imaging with CCD vs sCMOS sensors

For a detailed discussion of imaging sensors see J. Riba, Application of Image Sensors to Detect and Locate Electrical Discharges: A Review, Sensors, 2022.

CCD technology is the most commonly used sensor for Raman applications, which is unsurprising due to the high sensitivity and readout rates that can be achieved in spectroscopic applications. Although the spectrum to be recorded is dispersed across a rectangular or square array of pixels, the spectrum is obtained by vertical binning of pixels, a process in which the charge generated in each pixel by incident light in a column on the chip is combined by a stepwise transfer process before readout as a single data point in the resulting spectrum. With imaging, where each pixel is read out in sequence by vertical transfer (step shifting), readout artefacts can manifest in the image, such as shown in Figure S13. During the readout process, if the pixels remain under illumination then additional charge will be generated as the charge is shifted down the column and passes by through an illuminated pixel. This extra accumulation of charge can happen as the pixels on a CCD act both as converter of photon signals to electrical charge, as well as charge wells containing these generated electrical potentials. The accumulation of extra charges during readout leads to artefacts of constant intensity along the spatial axis. This is seen by the intensity plot from the CCD next to the vertical axis of the image. For normal readout modes such as full vertical binning (FVB) these artefacts are not usually of concern due to the binning of all vertical data points into a single value, but in imaging mode this effect has a significant impact when the exposure time is close the time needed for image readout.

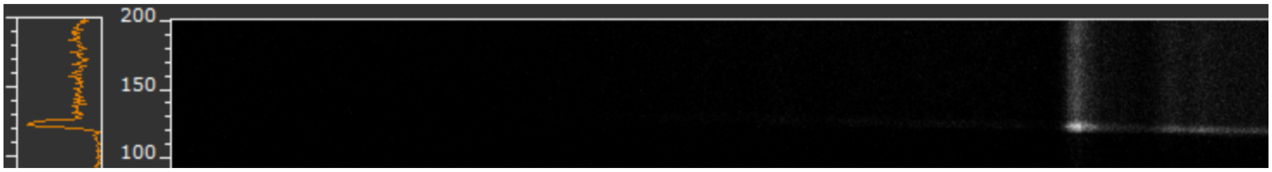

Figure S13 Intensity distribution of recorded image (orange graph, left) and image recorded with a 473 nm laser and an CCD detector in imaging mode (right). The intensity profile on the left of the image shows an artificially flat intensity profile at the top. This constant intensity profile is not due to constant laser power at the object plane, but rather due to the constant charge transfer rate between coupled vertical pixels.

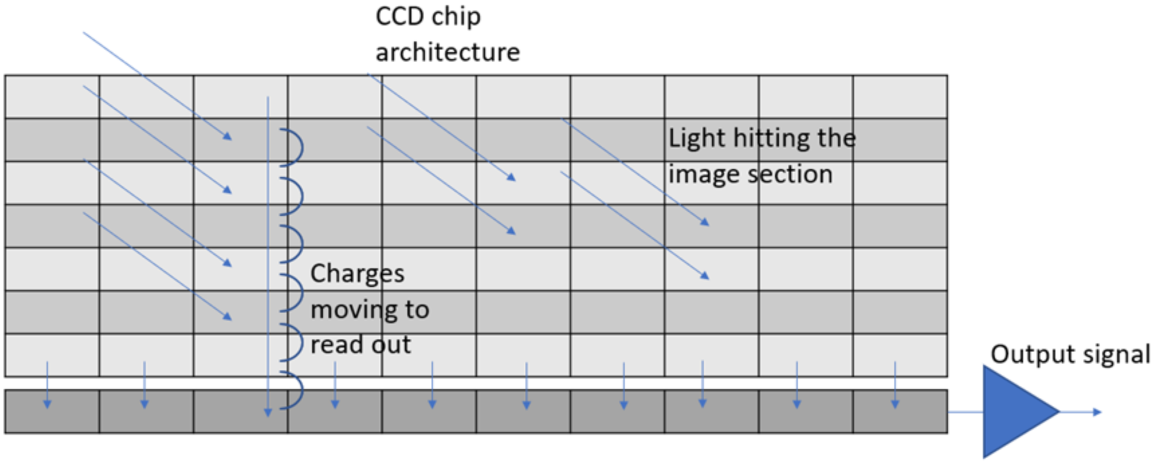

Figure S14 CCD chip architecture with vertical readout.

The observed intensity distribution seen in the image from the CCD is not expected for a point laser source. The shoulder of constant intensity in the oragne intensity graph is due to smearing during the CCD readout. The intensity of the streak relative to the rest of the image depends on the average intensity of light hitting the illuminated area and the readout rate of the detector, the speed at which the pixels are passing their charge. These are both constant numbers and hence we observe a shoulder of constant intensity. When spatial resolution on the vertical axis is required then images without such artefacts are needed and a shutter needs to be used to block additional illumination during readout. However, while keeping the shutter closed for readout results in much clearer images (Figure S2). Shuttering increases the measurement time significantly and is as such at the cost of overall image readout rate.

Images recorded using a camera with an sCMOS sensor can be obtained with fast readout rates; up to 100 Hz. Since each pixel has its own readout electronics and is read out individually, a physical shutter is not required and smearing is not observed.

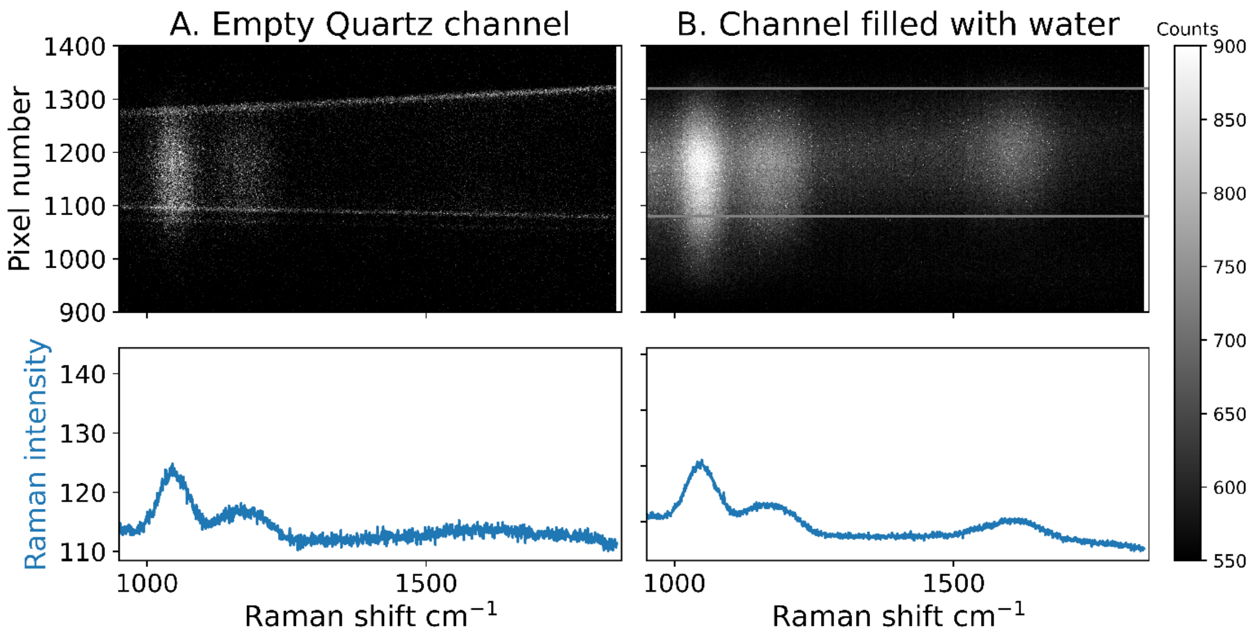

Figure S15 Quartz microreactor,(left) empty, and (right) filled with reagents in the laminar flow regime. Only the band of water is observed due to low concentrations of reagents used, and the lack of resonance enhancement of the Raman scattering of the reagents. Images were recorded at 473 nm with an sCMOS detector in imaging mode, orthogonal to the direction of flow. Lines become wider due to Petzval field curvature. Spectra obtained following full vertically binned of each image are shown below.

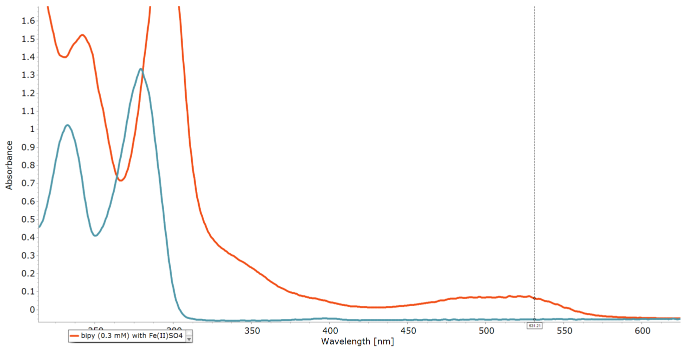

Figure S16 UV/vis absorption spectra of aqueous solutions of (green) bipy, and (red) a 3:1 mixture of bipy and Fe(II)SO_4 in water in a 1 cm pathlength quartz cuvette.
